# Asthma and Infertility: A Prospective Case–Control Study on Pregnancy and Live Birth Rates in Women With Asthma Undergoing Assisted Reproduction

**DOI:** 10.1111/crj.70180

**Published:** 2026-03-05

**Authors:** Frida E. Lundberg, Amalie H. Sneckenborg, Hanna Nilsson, Kenny A. Rodriguez‐Wallberg

**Affiliations:** ^1^ Department of Oncology‐Pathology, Laboratory of Translational Fertility Preservation Karolinska Institutet Stockholm Sweden; ^2^ Department of Reproductive Medicine, Division of Gynecology and Reproduction Karolinska University Hospital Stockholm Sweden

**Keywords:** assisted reproductive technology, asthma, clinical pregnancy rate, infertility, live birth rate

## Abstract

**Background:**

Previous studies have suggested a negative impact of asthma on fertility, and it has been hypothesized that the underlying inflammation is playing an important role. However, available data are scarce and mostly refer to women conceiving naturally. With this study, we wished to investigate whether asthma affects the outcomes of medically assisted reproduction or not.

**Methods:**

We conducted a prospective cohort study of 809 women, with (*N* = 213) or without asthma (*N* = 596), undergoing fertility treatment between 2009 and 2019 at one academic‐based reproductive medicine center. Treatment outcome measures included clinical pregnancy rate (CPR) and live birth rate (LBR) per stimulation cycle, per oocyte pickup, and per embryo transfer, among women undergoing in vitro fertilization treatment, and LBR among women treated by intrauterine insemination. Odds ratios and 95% confidence intervals were estimated using multivariable generalized estimating equation models.

**Results:**

No significant differences were found in CPR or LBR between women with asthma and women without asthma undergoing in vitro fertilization (*N* = 694) or intrauterine insemination (*N* = 45). In women with asthma, the adjusted odds ratio of clinical pregnancy per embryo transfer for all cycles was 0.91 (95% CI, 0.71–1.16), and the adjusted odds ratio of live birth per embryo transfer for all cycles was 0.90 (95% CI, 0.70–1.16). The adjusted odds ratio of live birth per performed intrauterine insemination was 1.42 (95% CI, 0.63–3.21) in women with asthma.

**Conclusions:**

Infertile women with asthma undergoing medically assisted reproductive treatment have a similar chance of achieving clinical pregnancy and live childbirth when compared to infertile women without asthma.

## Graphical abstract

This large prospective cohort study shows no impact of asthma on pregnancy and live birth rate among infertile women undergoing assisted reproduction.

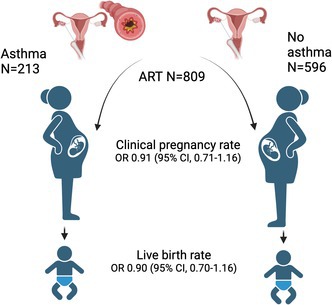

## 1 Introduction

Fertilization through assisted reproductive technologies (ART) is becoming more and more common, and nearly 5% of all children in the Nordic countries are conceived through ART [1, 2]. The pregnancy rate per cycle of in vitro fertilization (IVF), either using conventional IVF or intracytoplasmic sperm injection (ICSI), is around 30% [2, 3]. Intrauterine insemination (IUI) is less invasive than IVF and ICSI, with a lower pregnancy rate between 10% and 20% [4]. There are multiple reasons why most of the IVF, ICSI and IUI cycles fail; however, among the known factors, inflammatory response is suggested to play an important role [5, 6]. IUI is not currently accounted as ART but is encompassed in the broader term: medically assisted reproductive treatment.

Asthma is a chronic inflammatory disease that has been estimated to affect 3.2% of the adult population globally, with great variations in incidence and disease control between countries [7]. In a Swedish study from 2019, the prevalence of asthma was approximately 10% in women of childbearing age [8]. Asthma is characterized by hyperresponsiveness and bronchial obstruction and by infiltration of inflammatory cells and mediators in the inflamed tissue [9]. Inflammation caused by asthma can be systemic, and, although data are scarce, there are reports implying a negative influence on fertility [10, 11, 12, 13, 14]. Most available studies have either investigated the association between asthma and the number of offspring or asthma and time to pregnancy [13]. Although results are in part conflicting, the results suggesting an association between asthma and reduced fertility are further underpinned by evidence of an association between asthma and an increased utilization of ART [14, 15, 16, 17]. Yet, knowledge is still very scarce on whether or not asthma may also affect ART treatment outcomes. In a single study from 2016, a longer time to pregnancy and a lower pregnancy rate were observed in 96 women with a diagnosis of asthma undergoing fertility treatment when compared to infertile controls without asthma [18].

The aim of this study was to further investigate clinical pregnancy rate (CPR) and live birth rate (LBR) following IVF, ICSI, or IUI in women with asthma compared to a control group of infertile women without asthma.

## 2 Methods

**FIGURE 1:**
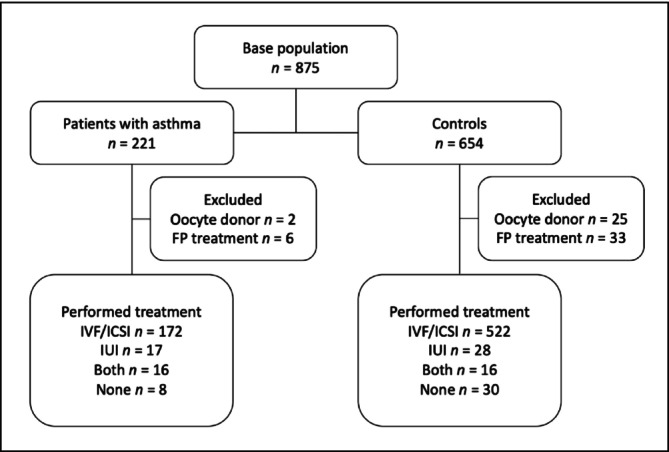
Flowchart of study population and their treatments during 2009–2019. FP = fertility preservation, ICSI = intracytoplasmic sperm injection, IUI = intrauterine insemination, IVF = in vitro fertilization.

The prospective study population included 875 infertile women who all underwent ART treatment at the Reproductive Medicine Unit of Karolinska University Hospital between January 1, 2009, and December 31, 2019. A total of 221 women with asthma and 654 healthy controls (no known comorbidities) were included. When possible, three controls per case were matched per treatment year. Controls were extracted from the second half of the year (June 1 to December 31) to avoid individuals using anti‐allergic drugs, which are common during the spring season. After exclusion of treatments using donor eggs or aimed at fertility preservation, the study cohort included 809 patients: 213 women with asthma and 596 controls (Figure 1). Study data were retrieved from the clinic through the clinical registry Linnéfiler (Fertsoft AB, Uppsala, Sweden).

The primary outcome measures for IVF and ICSI treatments were CPR and LBR following a fresh stimulation cycle and cumulative CPR and LBR rates following fresh and frozen–thawed cycles combined. The rates were calculated per initiated stimulation cycle, per oocyte pickup (OPU), and per embryo transfer (ET). Clinical pregnancy was defined as a viable intrauterine pregnancy confirmed by transvaginal ultrasound in the seventh gestational week. Outcome measures for IUI were LBR per planned and performed insemination. Secondary outcomes for IVF and ICSI included dose of follicle‐stimulating hormone (FSH), number of oocytes retrieved, fertilization rate, ovarian sensitivity index (OSI; defined as total FSH per oocytes retrieved), and proportion of single embryo transfer (SET). Potential confounders were decided a priori from variables with known impact on ART outcomes and included age at treatment, calendar year of treatment, body mass index (BMI), previous pregnancies, and childbirth before ART treatment.

## 3 Statistical Analysis

Continuous variables were presented as means with a 95% confidence interval (CI) and *p*‐values were calculated using the student's *t*‐test and chi‐square test of proportions. Odds ratios (OR) with 95% CIs of LBR and CPR were estimated using logistic regression models with cluster‐robust standard errors to account for dependencies between repeated treatments for each patient. The outcomes were analysed per started cycle, per OPU and per ET of IVF/ICSI, and per planned and performed cycle of IUI. Associations between asthma and each of the outcomes were assessed in univariable and multivariable models adjusting for age at treatment initiation, BMI, calendar period of treatment, previous pregnancy, and childbirth. Models per embryo transfer were also adjusted for number of embryos transferred. A sensitivity analysis, excluding women with other diseases in addition to asthma, was conducted as described above. Stata Version 17 (StataCorp. 2021. *Stata Statistical Software: Release 17*. College Station, TX: StataCorp LLC) was used for data preparation and statistical analyses. All statistical tests were two‐sided with a significance level of 0.05. The study follows the Strengthening the Reporting of Observational Studies in Epidemiology (STROBE) reporting guideline.

## 4 Results

### 4.1 Patient Characteristics

During the 10‐year inclusion period, a final cohort of 213 women with asthma was compared to 596 healthy controls. Most patients underwent IVF/ICSI: 172 cases and 522 controls. Additionally, 17 women with asthma and 28 controls underwent IUIs. Sixteen patients in each group underwent both IVF/ICSI and IUI (Figure 1).

The patient characteristics for women with asthma did not differ substantially from the controls with regard to age, year of inclusion, number of previous pregnancies, or number of previous children (Table 1). However, women with asthma were more likely to be overweight compared to the control group; 44% of the patients with asthma were in the BMI category > 24.9 kg/m^2^, compared to 35.2% in the control group, impacting the expected distribution (*p* = 0.016). The most marked difference between the groups was in the underlying infertility diagnosis (*p* = < 0.01). A greater proportion of patients with asthma underwent IVF/ICSI or IUI treatment as single without partner: 17.4%, versus 4.9% in the control group. In 10.8% of patients with asthma and 36.6% of the control group, the cause of infertility was not specified (Table 1).

**TABLE 1:** Patient characteristics.

	Asthma		Controls		*p*
	*N*	%	*N*	%	
Total	213	100	596	100	
Age					
< 25 years	1	0.5	21	3.5	*p* = 0.06
25–29 years	44	20.7	106	17.8	
30–34 years	89	41.8	225	37.8	
35–42 years	79	37.1	244	40.9	
Year					
2009–2010	38	17.8	125	21.0	*p* = 0.14
2011–2013	59	27.7	189	31.7	
2014–2016	59	27.7	121	20.3	
2017–2019	57	26.8	161	27.0	
Body mass index (BMI)					
< 18.5	5	2.4	18	3.6	*p* = 0.016
18.5–24.9	112	53.6	310	61.3	
25.0–29.9	54	25.8	117	23.1	
≥ 30.0	38	18.2	61	12.1	
Infertility diagnosis					
Ovulatory	29	13.6	65	10.9	*p* = < 0.01
Structural	4	1.9	22	3.7	
Endometriosis	9	4.2	34	5.7	
Genetic	21	9.9	48	8.1	
Male factor only	37	17.4	81	13.6	
No male partner	37	17.4	29	4.9	
Other or unexplained	53	24.9	99	16.6	
Unspecified diagnosis	23	10.8	218	36.6	
Previous pregnancies					
0	36	16.9	136	22.8	*p* = 0.13
1	82	38.5	246	41.3	
2	48	22.5	121	20.3	
3	24	11.3	50	8.4	
≥ 4	23	10.8	43	7.2	
Previous children					
0	70	32.9	231	38.8	*p* = 0.23
1	108	50.7	287	48.2	
≥ 2	35	16.4	78	13.1	

### 4.2 Treatment Characteristics

The length of hormonal simulation (days) did not differ between the groups. Stimulation using gonadotropins was used in all cases except for a few cases of oral treatments for ovulation induction (three in women with asthma and five in controls) (Table 2). The total FSH dose per stimulation was significantly lower in women with asthma, 2383 IU, versus controls, 2524 IU (*p* = 0.013). Both the mean number of oocytes at OPU and the OSI, reflecting the ovarian sensitivity to gonadotropins, were similar between the two groups. No significant differences were found between the groups in terms of fertilization rate (%) or the number of embryos transferred (Table 2).

**TABLE 2:** Treatment characteristics.

	Asthma	Controls	*p*
Days of gonadotropin^a^	10.6 (10.4–10.9)	10.7 (10.6–10.9)	0.538
Daily FSH dose (IU)^a^	219 (208–231)	239 (232–247)	0.006
Total FSH dose (IU)^a^	2383 (2232–2533)	2621 (2524–2718)	0.013
Oocytes at OPU (*n*)	9.38 (8.71–10.06)	9.03 (8.61–9.45)	0.399
OSI (total FSH/oocyte retrieved)	530 (475–586)	465 (372–559)	0.241
Fertilization rate (%)	65.8 (62.8–68.8)	63.3 (61.4–65.1)	0.162
Embryos transferred (*n*)	1.16 (1.10–1.21)	1.21 (1.18–1.24)	0.102

*Note:* Data presented as mean (95% CI), difference of means and *p*‐values calculated using Student's *t*‐test.

Abbreviations: CI = confidence interval, ET = embryo transfer, FSH = follicle stimulation hormone, IU = international units, OPU = oocyte pickup, OSI = ovarian sensitivity index.

^a^ In cycles with gonadotropin stimulation.

A SET was performed in the majority of treatments: 91% of the ETs in the asthma group and 87% in the control group. In the asthma group, 673 cycles IVF/ICSI led to 130 clinical pregnancies and 119 live born children. In the control group, 1691 cycles IVF/ICSI led to 353 clinical pregnancies and 328 live born children (Table 3).

**TABLE 3:** Clinical pregnancy rate (CPR) and Live birth rate (LBR) in women with asthma versus controls.

	Asthma events	Asthma cycles	Control events	Control cycles	OR^a^ (95% CI) Model I: Adjusted for age	OR^a^ (95% CI) Model II: Further adjusted^b^
IVF and ICSI						
Clinical pregnancy						
Per stimulation						
Fresh cycles	73	305	207	887	1.03 (0.74–1.41)	1.08 (0.74–1.57)
All cycles	130	673	353	1691	0.89 (0.70–1.14)	0.95 (0.73–1.23)
Per OPU						
Fresh cycles	73	291	207	843	1.02 (0.74–1.41)	1.09 (0.75–1.60)
All cycles	130	659	353	1646	0.88 (0.69–1.13)	0.95 (0.74–1.24)
Per ET						
Fresh cycles	73	228	207	650	1.02 (0.72–1.44)	1.10 (0.73–1.65)
All cycles	130	466	353	1149	0.88 (0.67–1.14)	0.98 (0.74–1.30)
LBR						
Per started						
Fresh cycles	68	305	194	887	1.02 (0.73–1.42)	1.07 (0.73–1.57)
All cycles	119	673	328	1691	0.87 (0.67–1.12)	0.91 (0.70–1.19)
Per OPU						
Fresh cycles	68	291	194	843	1.01 (0.72–1.42)	1.09 (0.74–1.61)
All cycles	119	659	328	1646	0.86 (0.67–1.12)	0.92 (0.70–1.20)
Per ET						
Fresh cycles	68	228	194	650	1.01 (0.71–1.44)	1.11 (0.73–1.69)
All cycles	119	466	328	1149	0.86 (0.65–1.13)	0.95 (0.71–1.28)
IUI						
LBR						
Per planned	18	121	15	142	1.40 (0.67–2.95)	1.20 (0.52–2.76)
Per performed	18	90	15	95	1.33 (0.58–3.06)	0.89 (0.38–2.07)

Abbreviations: CI = confidence interval, ET = embryo transfer, ICSI = intracytoplasmic sperm injection, IUI = intrauterine insemination, IVF = in vitro fertilization, LBR = live birth rate, OPU = oocyte pickup.

^a^ Odds ratio with a 95% confidence interval with controls as reference for all comparisons.

^b^ Adjusted for age, BMI, calendar period, previous pregnancy, and previous childbirth. Models per embryo transfer also adjusted for number of embryos transferred.

### 4.3 CPR

There was no significant difference in CPR per started fresh cycle between women with and without asthma (adjusted OR 1.08, 95% CI 0.74–1.57). Similarly, no significant difference in CPR was found when including both fresh and frozen/thawed cycles (adjusted OR 0.95, 95% CI 0.73–1.23). Results were similar when CPR was calculated per OPU and per ET (Table 3).

### 4.4 LBR

There was no significant difference in LBR following IVF/ICSI between women with and without asthma, neither for fresh cycles (adjusted OR 1.07, 95% CI 0.73–1.57) nor for fresh and frozen/thawed cycles combined (adjusted OR 0.91, 95% CI 0.70–1.19). Similarly, the LBR per OPU or ET was not significantly different. There was no significant difference in LBR per performed IUI treatment in women with and without asthma (adjusted OR 0.89, 95% CI 0.38–2.07) (Table 3).

After a sensitivity analysis excluding women with asthma in combination with known comorbid conditions (*n* = 48), the main results did not change (Table S1).

## 5 Discussion

This prospective cohort study has investigated the outcomes of IVF/ICSI and IUI treatment in women with asthma and compared them to outcomes in women without asthma. A cohort was prospectively identified over a period of 10 years and adjusted for appropriate confounders. No association was found between asthma and the outcomes of ART treatments in this infertile cohort, indicating that the chances of clinical pregnancy and live birth through ART are maintained in women diagnosed with asthma and comparable to controls without asthma. To our knowledge, this is the largest prospective study to date and adds to the scarce data investigating the potential association between asthma, LBR, and CPR following ART treatment.

Gade et al. have previously reported on 245 women with unexplained infertility undergoing IVF, ICSI, or IUI treatment and observed an increased time to pregnancy for women with asthma compared to women without asthma [18]. In our study, we did not consider time to pregnancy but instead the standardized rates of pregnancy and LBR. The rates of pregnancy per embryo transfer or per insemination in the women in our study were all within the expected range and did not differ between the groups (unadjusted PR 32% vs. 31.8% for IVF/ICSI and 20% vs. 15.7% for IUI in women with or without asthma, respectively). However, it is noteworthy to discuss several studies suggesting an association between asthma and infertility [13, 14, 15]. Hansen et al. concluded that women with asthma are more likely to conceive children through ART compared to the general population [15]. These results are supported by Jöud et al., whose population‐based study indicated increased risk of infertility, increased pregnancy loss, and increased induced abortions as well as increased utilization of ART in women with asthma [14]. Both studies point towards an association of infertility with asthma. In our study, we included only women with infertility all requiring medical assistance to reproduction, and we could evaluate the efficacy of the treatments. We found no impact on PR or LBR per ET in our infertile cohort. Other studies have suggested that inflammation itself plays a key role in the process of implantation [17], but in all, studies implying a direct impact of inflammation on ART are scarce. In a case–control study by Feichtinger et al., no significant differences were found in pregnancy rate, LBR, or estimates of implantation in women with endometriosis‐associated infertility, which is considered a reproductive disease with inflammatory features [19]. Similarly, Friedman et al. found no significant association between LBR in women with inflammatory bowel disease undergoing ART treatment, except after surgery [20].

A limitation of our study is that the control group was obtained by selecting controls based on calendar period and not through random selection. This limitation may cause a biased distribution of unmeasured confounding between the two groups. The rationale to select controls from the second part of the years' treatments at the clinic was to avoid seasonal allergies during the spring and anti‐allergic medications in the otherwise healthy infertile women. The women with asthma could also have other comorbidities; however, a sensitivity analysis excluding all comorbidities suggested that this did not influence the results. Further limitations of this study are the lack of information on pharmacological treatments for asthma, disease severity, cigarette smoking, and socioeconomic status. Although this study was not designed to assess the impact of asthma medication, Grzeskowiak et al. concluded that management of asthma with inhaled corticosteroids alone or in combination with short‐acting β‐agonists was associated with increased time to pregnancy, possibly underpinning the results observed by Gade et al. [16, 18]. Smoking cigarettes has a known negative effect on reproductive health, and studies have suggested an association between ART outcomes and smoking habits [21, 22]. In 2019, a Swedish report estimated that approximately 13% of the adult women with asthma smoked daily, although the prevalence of smoking at registration to antenatal care has decreased from around 9% to 4% during the study period [23, 24]. Women with higher socioeconomic status undergo more ART treatments and have a higher treatment success rate than women with a lower socioeconomic status even in the Nordic countries where infertility treatment is tax‐funded [25, 26]. There is also a known association between asthma and lower socioeconomic status [27]. Whereas all women with asthma seeking fertility care were included in the study cohort, women with lower socioeconomic status, and possibly more severe asthma, may not be as likely to seek care, hence affecting the generalizability of our results.

## 6 Conclusion

In this large prospective cohort study, we found no adverse association between asthma and the outcomes of medically assisted reproductive treatments. Women with asthma who undergo IVF and ICSI appear to have similar chances of a clinical pregnancy as women without asthma who undergo comparable treatments. Additionally, the LBR following IUI, IVF, and ICSI did not differ between women with and without asthma. The results are reassuring for women with asthma in need of treatment for infertility.

## Author Contributions

F.E.L. and K.A.R.‐W. are responsible for conceptualization and methodology. A.H.S., H.N., K.A.R.‐W., and F.E.L. compiled and interpreted the results, and K.A.R.‐W., F.E.L., H.N., and A.H.S. wrote and revised the manuscript. All authors have read and agreed upon the final manuscript and approved the submitted version.

## Funding

This study was supported by research grants from Cancer Research Foundations of Radiumhemmet (201313), the Swedish Research Council (2021‐06116), the Swedish Cancer Society (Dnr 20 0170F), Stockholm County Council, and Karolinska Institutet to K.A.R.‐W.

## Ethics Statement

The Regional Ethics Committee in Stockholm approved the study (Dnr 2011/1758‐31/2, amendments 2014/1825‐32 and 2018/275‐32).

## Conflicts of Interest

The authors declare no conflicts of interest.

## Supporting information


**Table S1:** Sensitivity analyses for clinical pregnancy rates (CPR) and live birth rate (LBR) in patients with asthma versus controls after excluding women with comorbidities*.

## Data Availability

Individual participant data are unavailable. Any request for data access should be directed to kenny.rodriguez-wallberg@ki.se.
