# Supplementary material for: Asthma and Infertility: A Prospective Case–Control Study on Pregnancy and Live Birth Rates in Women With Asthma Undergoing Assisted Reproduction
Source: Clin Respir J. 2026 Mar 5;20(3):e70180. doi: 10.1111/crj.70180 (PMC12963455; doi:10.1111/crj.70180)
Supplement: Supplementary file 1 — Table S1: Sensitivity analyses for clinical pregnancy rates (CPR) and live birth rate (LBR) in patients with asthma versus controls after excluding women with comorbidities*. [file CRJ-20-e70180-s001.docx]

Supplementary table 1. Sensitivity analyses for clinical pregnancy rates (CPR) and live birth rate (LBR) in patients with asthma versus controls after excluding women with comorbidities*

|  | | **Asthma**  **Events** | **Asthma**  **cycles** | **Control**  **events** | **Control**  **cycles** | **OR^1^ (95% CI)**  **Model I: Adjusted for age** | **OR^1^ (95%CI )**  **Model II: Further adjusted^2^** |
| --- | --- | --- | --- | --- | --- | --- | --- |
| **IVF and ICSI** | |  |  |  |  |  |  |
| **Clinical Pregnancy** | |  |  |  |  |  |  |
| Per stimulation | |  |  |  |  |  |  |
|  | Fresh cycles | 54 | 235 | 207 | 887 | 0.97 (0.67-1.38) | 1.05 (0.70-1.58) |
|  | All cycles | 97 | 543 | 353 | 1691 | 0.81 (0.61-1.06) | 0.87 (0.65-1.15) |
| Per OPU | |  |  |  |  |  |  |
|  | Fresh cycles | 54 | 223 | 207 | 843 | 0.97 (0.67-1.39) | 1.07 (0.71-1.62) |
|  | All cycles | 97 | 531 | 353 | 1646 | 0.80 (0.61-1.06) | 0.87 (0.65-1.16) |
| Per ET | |  |  |  |  |  |  |
|  | Fresh cycles | 54 | 181 | 207 | 650 | 0.92 (0.63-1.35) | 1.00 (0.64-1.55) |
|  | All cycles | 97 | 377 | 353 | 1149 | 0.79 (0.59-1.05) | 0.89 (0.66-1.22) |
| **LBR** | |  |  |  |  |  |  |
| Per started | |  |  |  |  |  |  |
|  | Fresh cycles | 52 | 235 | 194 | 887 | 1.00 (0.69-1.44) | 1.07 (0.71-1.63) |
|  | All cycles | 89 | 543 | 328 | 1691 | 0.79 (0.60-1.05) | 0.83 (0.62-1.11) |
| Per OPU | |  |  |  |  |  |  |
|  | Fresh cycles | 52 | 223 | 194 | 843 | 1.00 (0.69-1.45) | 1.10 (0.72-1.68) |
|  | All cycles | 89 | 531 | 328 | 1646 | 0.79 (0.59-1.04) | 0.83 (0.62-1.12) |
| Per ET | |  |  |  |  |  |  |
|  | Fresh cycles | 52 | 181 | 194 | 650 | 0.96 (0.65-1.41) | 1.04 (0.66-1.64) |
|  | All cycles | 89 | 377 | 328 | 1149 | 0.77 (0.57-1.04) | 0.86 (0.62-1.19) |
| **IUI** | |  |  |  |  |  |  |
| **LBR** | |  |  |  |  |  |  |
| Per planned | | 14 | 93 | 15 | 142 | 1.47 (0.66-3.28) | 1.13 (0.43-2.97) |
| Per performed | | 14 | 68 | 15 | 95 | 1.50 (0.55-4.05) | 1.01 (0.36-2.83) |

^1 Odds ratio with a 95% confidence interval with controls as reference for all comparisons.^

^2 Adjusted for age, BMI, calendar period, previous pregnancy and previous childbirth. Models per embryo transfer also adjusted for number of embryos transferred.^

^Abbreviations: CI = confidence interval; ET = Embryo transfer; ICSI = Intracytoplasmic sperm injection; IVF =^ *^in vitro^* ^fertilization; IUI = intrauterine insemination; LBR = Live birth rate; OPU = Oocyte pick-up.^

^*Including Hypothyroidism, Hashimoto, depression, appendicitis, migraine, IBS, Thalassemia minor, Fibromyalgia, Endometriosis, retinoblastoma, eczema, polycystic kidneys, Ehlers Danlos, anxiety, celiac disease, ADHD.^
